# 

**Published:** 1871-12

**Authors:** 


### CLOSE OF THE VOLUME.

This number completes the twenty-fifth volume of the DENTAL REGISTER. A quarter of a century has passed away since its first number was issued, and within that time what a change has taken place, not only in the journal, but in the profession.

The REGISTER was, in the beginning, issued as a quarterly, by Dr. James Taylor, of this city, under the auspices of the Mississippi Valley Dental Society; and then, with its little handful of subscribers, no one entertained very sanguine expectations that it would have more than a brief career; but still, by the persevering effort and energy of its publisher and editor, it lived and became an institution for the profession—for the West especially, and to some extent for the profession of the whole country—and still without a suming to be cosmopolitan. The REGISTER has always been modest.

Fifteen years ago the proprietorship and editorial management of the REGISTER was vested in and devolved upon J. Taft and Geo. Watt. In 1860 the publication of the REGISTER was transferred to John T. Toland, who had it in charge for three years, after which its publication reverted to the present editor and proprietor. It was changed from a quarterly to a monthly in July, 1860, since which time it has been published without interruption as a monthly.

In all these years the aim has been to make the REGISTER subserve the best interests of the professio[illegible], and we trust that in the future it may accomplish its mission better than hitherto.

### SPECIAL NOTICES.

The next meeting of the Wisconsin State Dental Society will be held at Watertown, on the second Tuesday in January—9th—1872, commencing at 9:30 A. M.

*Officers of the Society.*—President, Dr. E. Palmer, of LaCrosse, 1st Vice President, Dr. J. C. Lukes, of Racine; Recording Sec. Dr. Chas. C. Chittenden, of Madison; Corresponding Sec., Dr. D. W. Perkins, of Milwaukee; Treas., Dr. A. Solliday, Watertown.

*Standing Committees.*—Ex. Com., Drs. R. S. Wells and L. C. Stewart; on Publication, Drs. C. C. Chittenden, C. W. Barns and Wm. Decker; on Membership, Drs. E. M. Clark, M. B. Johnson and E. G. Hazleton; on Dental Ethics, Drs. D. W. Perkins, N. H. Drew and F. A. Williamson.

Subjects of great importance will be presented for the consideration of the profession, and it is hoped that every progressive dentist in the State will come prepared to *impart* as well as receive ideas. If you have any difficulties in your practice, come and have them solved. If you have any novelties in your practice, present them for the instruction of others. If you have adopted any new modes of practice, either in operative or mechanical dentistry, come prepared to give the profession the benefit of your experience. If you are using instruments of peculiar value to you, bring them, that we may be profited by an examination of them.

A clinic will be held during one of the sessions, conducted by some of our best operators. By applying to Dr. Solliday, of Watertown, hotel accommodations can be secured at reduced rates. Dentists from other States are cordially invited to attend and take part in its discussions. An invitation is also extended to the physicians of the State. Dentists who are willing to take part in the clinics are requested to come prepared with their instruments. No special arrangements have been made for securing essays, but it is hoped that the Society will be favored with several from gentlemen of well-known writing ability.

Commuted railroad tickets can be secured for the return trip, from the committee at Watertown.

*Order of Business.*—1. Calling to order, by the presiding officer. 2. Roll call. 3. Reading of minutes. 4. Election of members. 5. Address by the President. 6. Reports of officers. 7. Reports of committees. 8. Election of officers. 9. Selection of place for next meeting. 10. Discussions, clinics, etc.; 1st, Filling teeth—

special points in the operation; 2d, Clinics; 3d, Causes of the degeneracy of teeth; 4th, Anæsthetics: Their present relation to dental practice; 5th, The propriety of a law to regulate the practice of dentistry; 6th, Mechanical Dentistry. 11. Unfinished and miscellaneous business. Adjournment.

R. S. WELLS,
L. C. STEWART,
Executive Committee.

### A NEW MACHINE.

We have for a short time been using "Morrison's Bur-Engine" or Lathe. It proves to be a very good machine indeed. Its mechanism is very perfect, and it runs smoothly; it is light, convenient, and easily handled, runs without noise, and has more power than is ever required in operations upon the teeth, and runs with equal facility to the right or left. The point can be given any desired direction. Upon the whole, we regard it as a most excellent appliance, and one that no dentist in full practice can afford to be without. T.

THE DENTAL REGISTER—A CHANGE.—The publication of the DENTAL REGISTER has been transferred to and will be conducted by Drs. Spencer & Moore, of the "Ohio Dental Depot." They will begin with the twenty-sixth volume, the first number of which will be issued January prox. All subscriptions and advertising bills for that volume will be paid to them.

They are authorized to receive and receipt for money due to J. Taft for subscriptions on past volumes. It is very desirable that all accounts on past volumes should be closed at once, as we need the money. J. TAFT.

ATTENTION.—We send bills in this number of the REGISTER to all in arrears, with the desire and expectation that they will receive prompt attention. If there are mistakes, explain, and let them be compromised or settled. If any can not pay just now, please let us know the fact, and what we may expect. If there are any who can never pay, please let us know that, too, that we may not be consoling ourself with vain hope, and then, perhaps, be disappointed some time when we could not bear it so well as now. J. TAFT.

NEW YORK

### College of Dentistry.

### Sixth Annual Session - - 1871-72.

**FACULTY:**

**WILLIAM H. ALLEN,**
Emeritus Professor of the Institutes of Dentistry.

**FANEUIL D. WEISSE, M. D.,**

Professor of Regional Anatomy and General Pathology.

**FRANK ABBOTT, M. D.,**
Professor of Operative Dentistry and Oral Surgery.

**ALEX. W. STEIN, M. D ,**
Professor of Histology, Visceral Anatomy and Physiology.

**F. LeROY SATTERLEE, M. D.,**
Professor of Chemistry, Materia Medica and Therapeutics.

**C. A. WOODWARD, D. D. S.,**
Professor of Mechanical Dentistry.

**D. W. WILLIAMSON, D. D. S.,**

Demonstrator of Operative Dentistry.

**J. BOND LITTIG, D. D. S.,**

Demonstrator of Mechanical Dentistry.

**C. F. W. BODECKER, D. D. S.,**
Assistant to the Professor of Chemistry, Materia Medica and Therapeutics.

Students may Matriculate at any time, as the Infirmary is open and free for regular students of the college to practice in, the entire year.

The regular course of lectures will commence on Monday, October 16, and continue until the 1st of March. Three hours of each day (except Saturdays) will be devoted to lectures, and four hours to Clinics and Practice at the chair and in the Laboratory, under the direction of the demonstrators.

The Infirmary consists of three large rooms, each seventy-five feet long, with an excellent light to operate by, furnished with operating chairs and tables, all arranged to the best advantage for the more perfect instruction of students. Patients are usually in attendance in great numbers.

**TICKETS FOR THE COURSE, - - $150.00,**

Matriculation Demonstrators and Diploma Fees included.

For further particulars address

**FRANK ABBOTT, M. D., Dean**

June 1,'71, 6 mos. **78 W. 21th St., NEW YORK.**

### New Orleans Dental College

*Cor. of Carondelet & Perdido Sts.*

### FOURTH SESSION—1870-71.

### *FACULTY.*

JAS. S. KNAPP, D. D. S.,
Professor of Theory and Practice.

ANDREW F. McLAIN, D. D. S., M. D.,
Professor of Institutes of Dentistry.

WM. S. CHANDLER, D. D. S.,
Professor and Clinical Instructor of Dental Surgery.

CHAS. E. KELLS, D. D. S.,
Professor of the Science of Dental Mechanism.

S. MINTER ANGELL, M. D.,
Professor of Anatomy and Physiology.

J. S. HARRISSON, A. M., M. D.,
Professor of Materia and Medical Jurisprudence.

JOHN G. ANGELL, D. D. S.,
Clinical Instructor of Dental Mechanism and
Adjunct Prof. Operative Dental Surgery.

S. P. CUTLER, M. D., D. D. S.,
Prof. of Chemistry, Metallurgy, Microscopy and Histology.

DEAN OF THE FACULTY, - - - - JAMES S. KNAPP, D. D. S
*Office, No. 15 Baronne Street, New Orleans.*

The Fourth Regular Course of Lectures of the New Orleans Dental College for the Session of 1870-71, will commence on the 15th of November, and continue four months.

The superior advantages of this location for a Dental College should not be forgotten by students who live in a colder climate, and who by coming here will avoid the severity and rigors of a Northern winter, and enjoy four months of comparatively mild and pleasant weather. To those in delicate health this is an inducement of no inconsiderable importance.

**Professor's Tickets, for full course, - - - - - - $100 00**

**Matriculation (paid once), - - - - - - - - - - - - - 5 00**

**Diploma Fee, - - - - - - - - - - - - - - - - - - - - 30 00**

Board and lodging can be procured as cheaply in New Orleans as any large city in this country.

For further information address the Dean of the Faculty,

J. S. KNAPP, D. D. S.
Office No. 15 Baronne St., New Orleans

Or the Secretary,

PROF. A. F. McLAIN, D. D. S., M. D.
Acting Dean, Box 834

New Orleans, May 21, 1870. May, '70.

### CONTENTS.

COMMUNICATIONS

### The Dental Register

### OF THE WEST.

*Issued Monthly, at $3 a Year in Advance.*

DEVOTED TO THE INTERESTS OF THE DENTAL PROFESSION.

EDITED BY J. TAFT AND G. WATT.

The volume commences in January and closes in December.

The Register being issued monthly is always fresh, therefore indispensable to the practitioner who desires to keep posted up with the new inventions and improvements which are daily developed. Neither expense nor effort will be spared to give value and variety to its contents. Articles will be illustrated with well executed engravings whenever they will add to the interest or assist in explanation.

Its extensive circulation and frequency of issue makes it one of the BEST DENTAL ADVERTISING MEDIUMS in the United States.

RATES OF ADVERTISING.

One page, 1 year, $50. Half page, 1 year, $30. Quarter page, or card, 1 year $20.

All advertising bills payable in advance, or at furthest after the issue of the first number containing the advertisement. All transient advertisements to be paid for in advance.

☞ CONTRIBUTIONS SOLICITED.

Address, J. TAFT, or "DENTAL REGISTER," Cincinnati Ohio.

Business Communications to

J. TAFT, Publisher,

No. 117 West Fourth St.

